# Waves of genomic hitchhikers shed light on the evolution of gamebirds (Aves: Galliformes)

**DOI:** 10.1186/1471-2148-7-190

**Published:** 2007-10-09

**Authors:** Jan Ole Kriegs, Andreas Matzke, Gennady Churakov, Andrej Kuritzin, Gerald Mayr, Jürgen Brosius, Jürgen Schmitz

**Affiliations:** 1 Institute of Experimental Pathology (ZMBE) University of Münster, Von-Esmarch-Str. 56, D-48149 Münster, Germany; 2 Department of Physics and Mathematics, Saint Petersburg State Institute of Technology, 26 Moskovsky av., St.-Petersburg 198013, Russia; 3 Forschungsinstitut Senckenberg, Division of Ornithology, Senckenberganlage 25, D-60325 Frankfurt am Main, Germany

## Abstract

**Background:**

The phylogenetic tree of Galliformes (gamebirds, including megapodes, currassows, guinea fowl, New and Old World quails, chicken, pheasants, grouse, and turkeys) has been considerably remodeled over the last decades as new data and analytical methods became available. Analyzing presence/absence patterns of retroposed elements avoids the problems of homoplastic characters inherent in other methodologies. In gamebirds, chicken repeats 1 (CR1) are the most prevalent retroposed elements, but little is known about the activity of their various subtypes over time. Ascertaining the fixation patterns of CR1 elements would help unravel the phylogeny of gamebirds and other poorly resolved avian clades.

**Results:**

We analyzed 1,978 nested CR1 elements and developed a multidimensional approach taking advantage of their transposition in transposition character (TinT) to characterize the fixation patterns of all 22 known chicken CR1 subtypes. The presence/absence patterns of those elements that were active at different periods of gamebird evolution provided evidence for a clade (Cracidae + (Numididae + (Odontophoridae + Phasianidae))) not including Megapodiidae; and for *Rollulus *as the sister taxon of the other analyzed Phasianidae. Genomic trace sequences of the turkey genome further demonstrated that the endangered African Congo Peafowl (*Afropavo congensis*) is the sister taxon of the Asian Peafowl (*Pavo*), rejecting other predominantly morphology-based groupings, and that phasianids are monophyletic, including the sister taxa Tetraoninae and Meleagridinae.

**Conclusion:**

The TinT information concerning relative fixation times of CR1 subtypes enabled us to efficiently investigate gamebird phylogeny and to reconstruct an unambiguous tree topology. This method should provide a useful tool for investigations in other taxonomic groups as well.

## Background

In parallel to the application of new analytical methods, the avian phylogenetic tree has undergone substantial changes in the past decades. But even today many branchings remain highly controversial, although it is widely accepted that modern birds fall into two major clades, (1) Palaeognathae, a clade comprising rheas, kiwis, ostrich, emus,   cassowaries, and tinamous and (2) Neognathae, comprising Galloanseres (fowl and waterfowl) and Neoaves (all other taxa) [1,2]. Within Galloanseres, Galliformes (gamebirds) are traditionally classified into five families: Megapodiidae (megapodes, brush turkey and allies), Cracidae (currassows, guans, and chachalacas), Odontophoridae (New world quails), Numididae (Guinea fowl), and Phasianidae (pheasants, peacocks, partridges, and allies) [3-6].

While virtually all studies identify megapodes and cracids as successive sister taxa of the remaining Galliformes, the branching orders of Odontophoridae, Numididae, and Phasianidae, with its presumed subfamilies Tetraoninae (grouses) and Meleagridinae (turkeys), are less clear. Especially, the interrelationships between Numididae, Odontophoridae, and Phasianidae are considered a "major puzzle" of galliform phylogeny [7]. Also debated are the exact affinities of Tetraoninae and Meleagridinae. In traditional classifications, these two taxa are separated from the other Phasianidae, whereas molecular sequence analyses support a position of Tetraoninae and Meleagridinae deeply within a clade including the other Phasianidae [8-11]. Recently, Kaiser et al. [12] investigated phylogenetically informative retropositions in Galliformes and found significant support for two clades, (I) a monophyletic Phasianidae including Meleagridinae and Tetraoninae and (II) a clade comprising Meleagridinae, Tetraoninae, *Phasianus*, and *Tragopan*. The relative positions of Numididae and Odontophoridae and the topology of the phasianid tree differed from other sequence-based studies depending on the genes investigated and the analytical methods used [9-11,13-18].

Discrepancies in phylogenetic reconstructions based on various paleontological, morphological, behavioral, and molecular methodologies are often due to the presence of homoplastic characters [19]. Markers that are less likely to be confounded by problems of homoplasy include rare genomic changes (RGC) such as random insertions and deletions (indels) and retroposed elements [20]. Indels are frequently used for phylogenetic reconstructions [19,21-24] and the presence/absence patterns of retroposed elements have proven invaluable for reconstructing virtually ambiguity-free phylogenetic trees [25-29]. Presence/absence data resemble virtually homoplasy-free multistate characters with an extremely large possible number of unique character states. Steel and Penny [30] suggest that for this kind of data, maximum parsimony converge to a maximum likelihood estimator. The clear "presence" of a retroposed element at orthologous positions in related taxa indicates a derived condition acquired via a common ancestor, while its "absence" in more distant taxa represents the plesiomorphic condition prior to integration. Retroposed elements contain several features that, on their own, are very unlikely to occur twice independently at orthologous genomic positions. These include defined subtypes of retroposed elements, diagnostic mutations, and characteristic truncations of the consensus retroelements. Although presence/absence patterns are virtually homoplasy-free, there does exist, as for any other marker system, a low probability of lineage sorting [20] and a slight chance of exact excision of retroposed elements with perfect direct repeats [31]. These caveats aside, a statistical framework was developed to evaluate presence/absence data [32], and presence/absence patterns of retroposed elements have now been successfully used to reconstruct, for example, the placental mammalian tree at the superordinal level [27], the monophyly of Cetartiodactyla [33] and Pegasoferae [34], the position of Primates within Supraprimates [29], and internal primate relationships [35-37].

In the chicken genome, retroposed elements of the chicken repeat 1 (CR1) family of Long INterspersed Elements (LINEs), with more than 200,000 copies, constitute 80% of all interspersed repeats and 3.1% of the entire genome [38,39], while the second largest fraction of retroposed elements, the Long Terminal Repeat elements (LTRs) of endogenous retroviruses, with 12,000 copies, constitute only 4.7% of all interspersed repeats [38,39]. As CR1 elements do not show target site duplication (direct repeats) [40,41], excisions, such as that described by van de Lagemaat [31], cannot occur, making them the most suitable retroposed elements in bird genomes for phylogenetic purposes [42].

**Figure 1:**
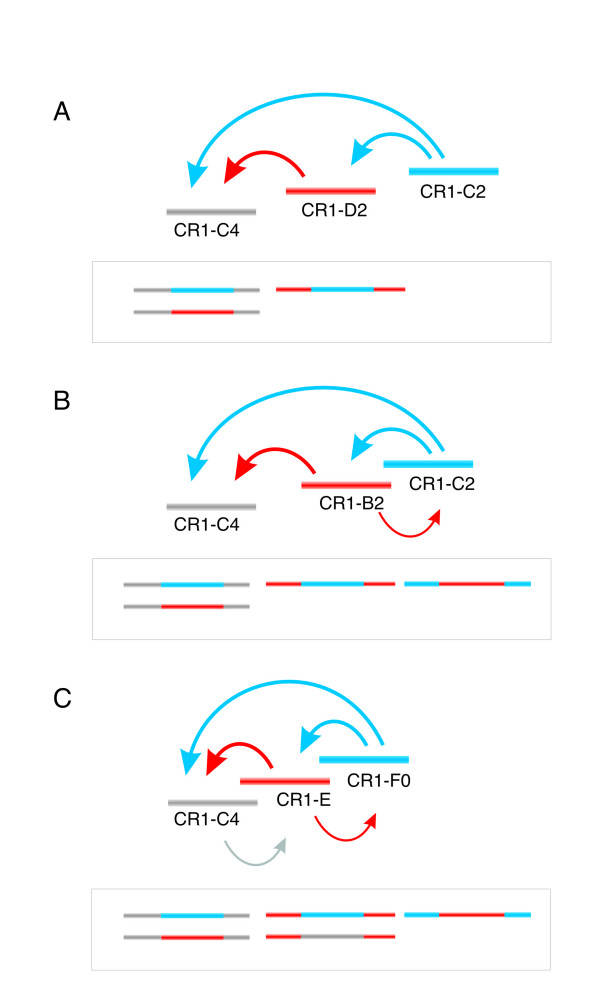
Principle behind the TinT method. Examples of directed insertions of CR1 elements active at different periods. (A) Shows three different CR1 subytpes, active at non-overlapping periods and their resultant TinTs (in box below). As indicated by blue arrows, the youngest element (C2) inserted into both older subtypes (D2 and C4). D2 was active after C4 became inactive and inserted into the latter (red arrow). (B) Example of CR1 subtypes active at overlapping and non-overlapping periods. Only elements that were active during overlapping periods (C2 and B2) had the opportunity to insert each into the other. As the activity period of the B2 element only partially overlapped that of the C2, fewer insertions occurred in the B2-C2 direction (indicated by the thinner arrow). (C) Example of three CR1 subtypes active at overlapping periods. Note that the activity of C4 does not overlap that of F0, thus there was no opportunity for C4 to insert directly into F0. Again, fewer insertions of older elements into younger ones are indicated by thinner arrows.

While full-length CR1 elements are 4.5 kb long and contain two open reading frames [41], most CR1 sequences are truncated copies of their autonomous full-length master genes [39,43]. CR1 subtypes are characterized by diagnostic mutations that occurred in their specific master genes. Different full-length copies of master genes remained transcriptionally active over long, overlapping, periods of time and distributed corresponding retroelements in specific waves of activity as has been described in penguins [44]. To efficiently select phylogenetically informative CR1 elements from the chicken genome, it would be helpful to know which CR1 elements were active at which evolutionary time points. As CR1 elements, like most other retroposed elements, integrate almost randomly into the genome, they also frequently insert into other CR1 copies. But, at a given point in time only the active CR1 subtypes can insert into copies of their own or other CR1 subtypes (Figure 1). This provides information about which 'host' CR1 subtypes were already integrated at this particular time point. If the reverse case, in which the 'host' subtype inserted into an active CR1 subtype, cannot be found in the entire genome, one can assume that the 'host' subtype was probably already inactive at this particular time point. Thus, an analysis of the patterns of nested CR1 elements (transpositions in transpositions that we call the TinT method, Figure 1) provides a relative timetable of active CR1 elements. A first step towards a genome wide characterization of the activity ranges of CR1 elements is to search for the distribution patterns of nested retroposons. Churakov et al. [45] recently applied a novel method based on the single-case patterns of nested retroposons to characterize the historical appearance of various armadillo-specific SINE subfamilies. Similarly, Ichiyanagi and Okada used this method to determine the full lengths of SINEs in zebrafish [46] and Pace and Feschotte to investigate DNA transposon activity in the human genome [47].

To further investigate and reconstruct the phylogenetic tree of Galliformes, we developed a multidimensional, computer applicable model for computing the frequencies of TinT genome-wide. Using this model, we describe the waves of activity or fixation patterns of various CR1 subtypes. We then used this information to directly select specific subtypes of retroposed elements that were active on the galliform evolutionary lineage leading to the chicken (*Gallus gallus*). These element subtypes were used to experimentally extract phylogenetic informative orthologous sequences from representative loci of all galliform families. As LTR elements also insert into each other, but were not frequent enough to apply the TinT method, we investigated their random insertions in the chicken genome for potential phylogenetic use. Furthermore, other phylogenetic signals (indels) observed during the genomic alignments of our presence/absence loci provided support for additional clades. With this multifaceted approach we reconstructed the major aspects of galliform evolution.

## Results and discussion

To obtain a relative temporal order of CR1 element activity for phylogenetic use, we explored the patterns of nested retroposed CR1 subtypes (Figure 1) from the chicken genome. From a genome wide collection of annotated retroposed elements we extracted all 1,978 cases of nested CR1 elements. The resulting matrix (additional data file 1) was used to calculate a multidimensional model (additional data file 1) giving the maximum probability of activity for each of the 22 CR1 subtypes on a relative timescale (TinT, see additional data file 1). The model makes the following assumptions: (i) For each CR1 subtype there was one limited period of activity. (ii) There was no known target site preference for the CR1 subtypes, thus each individual copy could have inserted at any random position in the genome. This could have been either an anonymous sequence or another CR1 copy. (iii) The number of copies of any given CR1 subtype in the genome reflects the duration of its activity. (iv) The temporal fixation rate of each CR1 subtype can be described by a normal distribution as is shown by the divergences of its single copies from their consensus sequences (additional data file 1). (v) The probabilities of fixation among the individual CR1 copies during their specific activity periods were relatively equal (equal promoter activity, equal affinity of reverse transcriptase to the mRNA, and an equal availability of reverse transcriptase). Based on these assumptions we developed a function describing the behavior of the fixation of each CR1 subtype on a relative timescale. Using the maximum likelihood approach we calculated the maxima of probability of fixation for each CR1 subtype.

**Figure 2:**
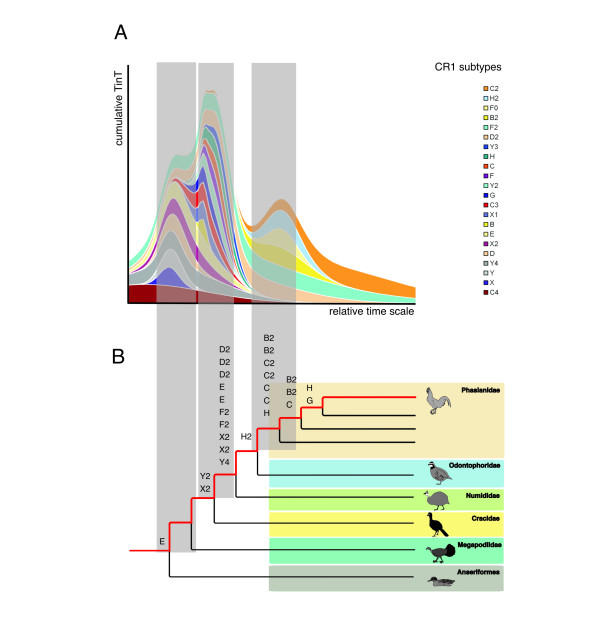
Comparison of TinT relative times of activity and the main divergences of the galliform tree. (A) Cumulative TinT of CR1 elements on a relative timescale during gamebird evolution. The graph shows the cumulative maximum probabilities of activities for nested CR1 retropositions that were fixed in the ancestral lineage of the chicken genome. (B) A simplified galliform tree showing the main divergences from the lineage leading to the chicken (in red). The CR1 subtypes depicted above the various branch points were identified by presence/absence analysis in species on the corresponding internal branches in this study and by Kaiser et al. [12]. The elements within the shadowed areas connecting (A) and (B) that were apparently active on specific branches of the galliform tree were dominating in corresponding peaks of the cumulative TinT graph.

The results show that the various CR1 subtypes differ not only in the median time points on the relative timescale during which they were actively fixed in the genome, but also in the lengths of these time windows, revealing three major peaks of concentrated CR1 activity/fixation (Figure 2A, additional data file 1). The oldest peak is dominated by CR1 subtypes Y4, D, X2, E, C4, Y, F2, D2, and X elements, the middle peak by CR1 subtypes Y2, C3, G, F2, X1, D2, H, Y4, E, and C, and the youngest peak by CR1 subtypes H2, F0, B2, F2, D2, and C2.

To verify the relative times of TinT activities we calculated the average divergencies of all CR1 subtypes from their consensus sequences. Assuming random accumulation of mutations, the degree of divergency should then be age-related. There was a significant correlation between the relative time scales of the cumulative TinT and the CR1 divergencies (*R *= -0,6489, *P *<< 0.01) (additional data file 1).

Armed with the relative ages of chicken CR1 subtypes deduced from the cumulative TinT, we selected a representative set of diverse CR1 elements, whose activity periods spanned the entire time frame of galliform evolution, to use as experimental probes of phylogenetic branch points of the other galliform species. Intronic sequences of the chicken genome (17,300 introns; maximal length of 1 kb) were screened for embedded CR1 elements and LTR elements (RepeatMasker) (300 CR1 and 45 LTR elements). These were inspected by eye in the genome browser (UCSC) [48] and the most conserved loci (120 cases), representing the broad activity range of CR1 subtypes as compiled by the TinT method, were chosen to generate conserved PCR primers for amplification of orthologous loci in representative galliforms (see Methods for species sampling). Of these, 19 loci were successfully amplified in all important taxa, revealing a total of 25 phylogenetically informative retroposed elements. CR1 elements present in these amplified loci, along with those presented by Kaiser et al. [12] show that at least the CR1 subtypes E, Y2, X2, Y4, F2, D2, H2, C, C2, B2, H, and G were active during galliform evolution. Moreover, the results of the cumulative TinT analysis are clearly in line with the exemplarily shown activities of certain CR1 elements identified by their presence/absence patterns in various species (Figure 2B). The elements represented in the older cumulative TinT peak we found as well to be active during the first divergences in galliform evolution, while the elements of the second peak were active during times of younger divergences. Thus the phylogenetic markers present an actual calibration of the TinT relative timescale. In further investigations we focused on screening for intronic CR1 elements with TinT-selected activity patterns in a galliform-wide amplification.

As our analysis of chicken sequences did not furnish elements that retroposed after the divergence of the lineage leading to turkeys from the one leading to the chicken, to provide phylogenetic information to solve the potential sister group relationships on this lineage we scanned the available turkey genomic trace sequences (about 6 million) for insertions of relatively young repeats (CR1-C2 and CR1-B2). Cases with elements absent in chicken orthologous genomic loci were selected in the genome browser (UCSC) [48] and we generated conserved PCR primers for eight loci.

**Figure 3:**
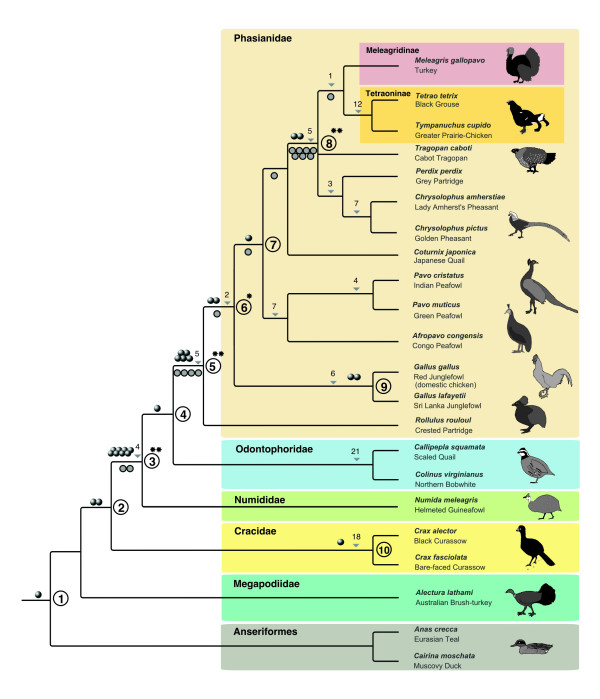
Retroposed elements as landmarks of galliform evolution. Gray balls represent single retroposition events revealed by our search for CR1 and LTR elements in chicken and turkey sequence databases. Filled gray circles denote markers published by Kaiser et al. [12]. Supported splitting points are labeled with Arabic numerals. Triangles denote branches supported by indel markers (amount given by numbers above) present in the same loci as the retroposed elements. The taxa shown represent only those from which we sampled CR1 elements and LTRs. Significantly supported splitting points are indicated with * *p *< 0.05, **p < 0.0001.

Altogether, we extracted a total of 23 orthologous loci containing 25 phylogenetically informative insertions of CR1 and LTR elements. The presence/absence patterns of these elements support ten clades within Galloanseres and Galliformes (Figure 3, additional data file 2): (1) One element (CR1-E) was found in species of Galliformes and Anseriformes but not in the outgroup zebra finch (*Taeniopygia guttata*; Passeriformes), thus supporting the clade Galloanseres. (2) Two elements (CR1-Y2 and CR1-X2) were found at orthologous genomic positions in all galliforms except Megapodiidae (megapodes). Although the pattern of the three possible branching orders – with the prior hypothesis at the first position – is not significant ([200]; *p *= 0.111) according to Waddell et al. [32], this is the first unambiguous retroposon evidence to support the local tree topology supported by morphological [16] and mitochondrial sequence data [10,15]. (3) We found eight independent retropositions (2× CR1-F2, 3× CR1-D2, 2× CR1-X2, 1× CR1-Y4) shared by Numididae, Odontophoridae, and Phasianidae that were not present in Anseriformes, Megapodiidae and Cracidae. Assuming a prior hypothesis based on morphological [16], nuclear [9], and mitochondrial sequence data [10,15], these markers, together with two insertions found by Kaiser et al. [12], provide statistically significant support ([10 0 0], *p *< 0.0001) for this branch of galliform evolution. (4) Odontophoridae and Phasianidae share one orthologous CR1-H2 insertion that is missing in Anseriformes, Megapodiidae, Cracidae, and Numididae. While morphological [16] and nuclear sequence data [9], as well as the combined data set of Crowe et al. [11] support a sister group relationship between these two taxa, mitochondrial analyses [10,15] support a topology in which Numididae are the sister taxon of Phasianidae. Our results provide the first, unambiguous retroposon evidence supporting the morphological and nuclear data. (5) We found five independent retroposon insertions (2× CR1-B2, 1× CR1-C2, 1× CR1-C and one GGLTR4a) in all Phasianidae species examined that were absent in all other galliform families. The monophyly of Phasianidae was also significantly supported by four additional independent CR1 insertions discovered recently by Kaiser et al. [12]. Thus the monophyly of Phasianidae (including Tetraoninae and Meleagridinae) is significantly supported by nine independent retroposition insertions ([9 0 0]; *p *< 0,0001). (6) One CR1-B2 and one CR1-C element were found in all investigated phasianids except *Rollulus rouloul*. Thus, together with the one additional insertion detected by Kaiser et al. [12], there is now significant support ([3 0 0]; *p *= 0.037) for a hitherto unexpected clade. (7) All investigated phasianids except *Rollulus *and *Gallus *were found to share a CR1-C retroposition. An additional independent CR1 insertion supporting the same topology ([2 0 0]; *p *= 0.11) was found by Kaiser et al. [12]. (8) We found two insertions of CR1-B2 elements in all investigated phasianids except the *Rollulus, Gallus, and Pavo *species. These findings lend additional support to the seven independent insertions found by Kaiser et al. [12] in similar species. The pattern of [9 0 0] is highly significant (*p *< 0.0001). (9) Two LTR (GGERVL18LTR) insertions were present in orthologous positions in *Gallus gallus *and *Gallus lafayetii *that were absent in all other analyzed birds. (10) One CR1-D2 insertion was found in *Crax alector *and *Crax fasciolata*, which was absent in all other investigated galliforms.

Within the loci containing the retroposed elements, we also found support for additional clades by the presence of 95 random intronic indels (see Figure 3). One indel was found only in the Meleagridinae and Tetraoninae species, a grouping that was also recently indicated by one CR1-insertion [12]. For example, twelve indels were exclusive to *Tetrao *and *Tympanuchus*, grouping these two species of the subfamily Tetraoninae together, three independent indels were specific for intronic sequences of *Perdix *and *Chrysolophus*, seven were unique to the two *Chrysolophus *species, seven were unique to a clade comprising *Pavo *and *Afropavo*, in agreement with Kimball et al. [49] and four were unique to *Pavo muticus *and *Pavo cristatus*. Twenty-one independent indels group together the two odontophorid genera *Callipepla *and *Colinus*, and eighteen unite *Crax alector *and *Crax fasciolata*. Together with all the retropositions presented in points 4, 5, 6, 7 and 8 above (Fig. 3), these data clearly support a sister group relationship between *Afropavo *and *Pavo*, rejecting an earlier morphology-based hypothesis of a clade comprising *Afropavo *and Numididae [50]. Although low complexity RGCs have a higher probability of being homoplastic [23] than do the insertion patterns of retroposons, we did not find any indels contradicting the topology of the tree supported by retroposed elements.

We provide the first retroposon evidence that Odontophoridae are the sister taxon of Phasianidae, which is also supported by at least one morphological feature, the presence of a well-developed intermetacarpal process on the carpometacarpus (see however, Stegmann (1978) concerning the possibility of a secondary loss of this process in Numididae [51]). Our study further provides clear evidence against a recently hypothesized sister group relationship between *Perdix *and Meleagridinae (Crowe et al. 2006; note that the morphological data set used in this analysis contains several incorrect character scorings [52]).

To the best of our knowledge, it has not yet been pointed out that the sister group relationship between New World turkeys and Palaearctic grouse, which is also supported by analyses of sequence data [11,14], indicates a New World origin of grouse. Because the clade (Meleagridinae + Tetraoninae) is nested within taxa with predominantly Asian distributions, the stem species of this clade probably reached the New World from Asia.

A striking observation from the TinT data is that the maximal frequencies of individual CR1 subtype fixation rates fall in close temporal proximity to one another and tend to be concentrated in distinct temporal waves, as is visible from the cumulative curve (Figure 2). Interestingly, two of these peaks of CR1 fixation rates coincide with the two most highly supported branches, indicating long internal branches and/or high retroposition fixation rates. At least five parameters might affect the fixation rate of retroposons in a population in a given branch of the phylogenetic tree: (I) the promoter activity of the master gene, (II) the overall availability of enzymatic retroposition machinery and (III) its affinity to the master RNA, (IV) the population size, and (V) the branch length. In small populations the fixation of a single retroposition event is much more likely than in large populations [53]. As the cumulative curve (Figure 2) reflects the timeframes of maximum fixation rates of several independent CR1 subtypes, it is unlikely that individual peaks are the result of promoter activity. The peaks might reflect times of over expression of the total retroposition machinery or might be due to severe population bottlenecks in the ancestral chicken lineage.

## Accession numbers

The GenBank accession numbers for the sequences discussed in this paper are [EU054465–EU054818].

## Conclusion

In summary, we present a method to calculate the relative times of maximum fixation frequencies for retroposons. We applied the TinT method to obtain a temporal order of the activity of different CR1 subtypes. The results of the TinT method enabled us to preselect potential phylogenetically informative presence/absence loci to test hypotheses for specific internal branches of a phylogenetic tree. With this preselection strategy, we found the first retroposition evidence supporting successive sister taxon relationships between Megapodiidae, Cracidae, Numididae, and the remainder of galliform birds. Highly significant support is presented for the first time for the monophyly of the phasianoid clade comprising Numididae, Odontophoridae and Phasianidae (Figure 3, point 3). One marker suggests that Numididae are the sister taxon of a clade comprising Odontophoridae and Phasianidae. Five independent retroposon insertions presented in this study, along with four previous ones, offer overwhelming support for the monophyly of all investigated phasianid species (Figure 3, point 5). We present the first significant support for a sister group relationship between *Rollulus *and all other investigated phasianids (Figure 3, point 6), and additional retroposon evidence for the monophyly of a clade containing *Pavo, Afropavo*, *Coturnix*, *Chrysolophus*, *Perdix*, *Tragopan*, *Tympanuchus*, *Tetrao *and *Meleagris *(Figure 3, point 7) and for a clade comprising *Chrysolophus, Perdix, Tragopan*, *Tympanuchus*, *Tetrao*, and *Meleagris *(Figure 3, point 8). Complementary information from random indels indicate the existence of a clade including *Tympanuchus*, *Tetrao*, and *Meleagris *another clade comprising *Tympanuchus *and *Tetrao*, one comprising *Chrysolophus *and *Perdix *to the exclusion of *Tragopan*, *Tympanuchus*, *Tetrao*, *Meleagris*, and the other investigated galliform taxa, and finally a clade comprising *Pavo *and *Afropavo *to the exclusion of all other investigated taxa.

The mathematical TinT model, applied to birds, is currently in the process of being tested in several mammalian groups. We believe that it will prove to be a significant tool for all genomic projects characterizing the activity periods of retroposed elements.

## Methods

### TinT method

We downloaded all 207,284 annotated genomic chicken CR1 sequences along with their 250 nt flanking regions from the University of California Santa Cruz (UCSC) Server [54,55]. We analyzed this dataset searching for internally retroposed element insertions using the local version of RepeatMasker [56]. From the RepeatMasker results we used a novel C-language script to extract all nested CR1 elements with their flanking CR1 host sequences. We considered a CR1 retroposed element to be nested if the upstream and downstream 25 nt flanking sequences were clearly assignable to CR1 host elements. We eliminated uninformative cases in which the host and nested CR1 elements were from the same subtype.

The number and subtype identity of all extracted host and nested CR1 elements were compiled in a 22-dimensional matrix (additional data file 1), which was used to calculate the relative integration period and transposition activity maxima for each nested CR1 subtype. This mathematical model (additional data file 1) considers the simplest scenario, that each CR1 subtype had only one period of activity and no specific target site preferences. For visualization we calculated cumulative TinT values (Figure 2).

### Average level of nucleotide divergency

An independent measure to examine the relative temporal order of transposon activity was obtained by comparing the average levels of nucleotide divergency from their consensus sequences in the various CR1 subtypes, assuming that the highest divergency appears in the oldest subtypes that became inactive first. The lowest divergency is expected for subtypes that are still active. However, the method is dependent on accurate consensus sequences [47]. As RepeatMasker calculates the nucleotide divergency of each retroposed element from its consensus sequence, we used these values to calculate the average subtype divergency level (additional data file 1). We then used linear regression analysis to correlate the divergency values to the relative temporal position of CR1 subtypes obtained by the TinT method.

### Computational strategies

We used four computational strategies to find phylogenetically informative loci featuring presence/absence patterns of retroposed elements and random indels.

#### Strategy I

An ideal starting point to investigate galliform phylogenetic relationships is the chicken, the domesticated form of the Red Junglefowl (*Gallus gallus*), because the genome of this model organism has been fully sequenced. Thus, retroposon insertions can be bioinformatically located and the orthologous loci can be experimentally investigated in other galliform species. Chicken intronic sequences (23,236 introns) were downloaded from the Santa Cruz Server [55,57]. After excluding duplicated sequences and, to facilitate PCR amplification, introns larger than 1 kb (17,300 introns), we screened for the presence of retroposed elements (RepeatMasker). The resulting loci (containing about 300 CR1 elements and 45 LTRs) were analyzed for the presence of conserved flanks (Santa Cruz Server) [58,59] and 120 loci containing elements that retroposed during different parts of the relative timescale (TinT) were chosen to generate PCR primers.

#### Strategy II

Because the turkey genome is currently being sequenced as well, preliminary genomic data can be used to locate retroposed elements and to experimentally investigate potential sister groups of turkey. We downloaded all available trace sequences (6 million) from the turkey (*Meleagris gallopavo*) genome [60] and searched for retroposed element insertions (RepeatMasker). As in strategy I, the resulting 784 loci were then analyzed for the presence of conserved flanks (Santa Cruz Server; [58]) and 8 loci containing copies of the relatively young CR1-B2 and CR1-C2 were chosen to generate PCR primers to investigate potential turkey sister groups.

#### Strategy III LTRs

The introns selected under strategy I that contained LTR insertions (45 loci) were analyzed for the presence of conserved flanks (Santa Cruz Server; [58]) and 7 loci containing LTR elements were chosen to generate PCR primers.

#### Strategy IV Random Indels

Although no concerted effort was made to conduct a systematic search for phylogenetically informative random indels, all alignments of our retroposon presence/absence markers were checked for this potential additional source of information. This was possible, because most galliform introns are highly conserved and thus more easily alignable, compared to many mammalian introns for example [27,28]. Here we restricted ourselves to shared indels larger than three nucleotides to ensure a certain level of complexity in the sequences, so that they would not be confused with coincidental random mutations.

### Taxon sampling

We analyzed DNA samples and/or sequences from the following bird species. Anseriformes: *Cairina moschata*, *Anas crecca*; Galliformes: *Alectura lathami*, *Crax fasciolata*, *Crax alector*, *Numida meleagris*, *Colinus virginianus*, *Callipepla squamata*, *Rollulus rouloul*, *Gallus lafayetii*, *Gallus gallus*, *Afropavo congensis*, *Pavo cristatus*, *Pavo muticus*, *Coturnix japonica*, *Perdix perdix*, *Chrysolophus pictus*, *Chrysolophus amherstiae*, *Tragopan caboti*, *Tetrao tetrix*, *Tympanuchus cupido*, *Meleagris gallopavo*, and the outgroup, *Taeniopygia guttata*.

### PCR amplification and sequencing

We designed PCR primers for sequences located in DNA regions highly conserved between chicken and human, to guarantee even higher conservation among the various galliform species (additional data file 2). PCR reactions were performed using Phusion DNA Polymerase (New England BioLabs, Beverly, MA). The first high throughput PCR was carried out in a 96-well plate format, amplifying the selected genomic loci from representatives of the investigated families and subfamilies: *Cairina moschata*, *Alectura lathami*, *Crax fasciolata*, *Numida meleagris*, *Callipepla squamata*, *Rollulus rouloul*, *Gallus gallus*, *Pavo cristatus*, *Tetrao tetrix*, and *Meleagris gallopavo*. PCR was performed for 30 s at 98°C followed by 35 cycles of 10 s at 98°C, 30 s at the respective primer-specific annealing temperature, and 30 s at 72°C. Following gel-electrophoreses, those loci in which fragment size shifts indicated the presence and/or absence of the embedded transposed elements were amplified in the expanded species sampling. All investigated PCR fragments were sequenced directly or purified on agarose gels, ligated into the pDrive Cloning Vector (Qiagen, Hilden), and electroporated into TOP10 cells (Invitrogen, Groningen). Sequencing was performed using the Ampli Taq FS Big Dye Terminator Kit (PE Biosystems, Foster City) and standard M13 forward and reverse primers.

### Statistical analyses

In our analyses of retroposition presence/absence data, we applied the statistical test developed by Waddell et al. [32] to determine the level of statistical support for particular branching points of the galliform phylogenetic tree. This methodology assumes the existence of a prior hypothesis based on other data, and calculates the relative probability that one of the three possible branching patterns is correct based on the number of independent retropositional markers supporting the various hypotheses. *p *< 0.05 was considered to be significant (usually achieved with a minimum of three independent retropositional insertions).

The results of the TinT method were compared to those of the relative timescale obtained by the average level of CR1 nucleotide divergency using the standard linear regression model.

## Authors' contributions

JOK, GC, and JS conceived and designed the experiments. JOK, AM, and GC performed the experiments, collected data, or did experiments for the study. JOK, AM, GC, AK, and JS analyzed the data. JB and JS contributed reagents/materials/analysis tools. JOK, GM, and JS wrote the paper.

All authors have read and approved the final paper.

## Supplementary Material

Additional file 1 contains information about the TinT method: (I) the probabilistic model, (II) the TinT matrix, (III) examples of divergency distributions of CR1 subtypes, (IV) TinT activity distributions for each element on a relative timescale, and (V) a comparison of results obtained by the TinT and divergency analyses. Click here for file

Additional file 2 contains a full presence/absence table of the phylogenetic analyses and a table of the PCR primers used to amplify informative loci. Click here for file
