# Supplementary material for: Waves of genomic hitchhikers shed light on the evolution of gamebirds (Aves: Galliformes)
Source: BMC Evol Biol. 2007 Oct 9;7:190. doi: 10.1186/1471-2148-7-190 (PMC2169234; doi:10.1186/1471-2148-7-190)
Supplement: Additional file 1 — contains information about the TinT method: (I) the probabilistic model, (II) the TinT matrix, (III) examples of divergency distributions of CR1 subtypes, (IV) TinT activity distributions for each element on a relative timescale, and (V) a comparison of results obtained by the TinT and divergency analyses. [file 1471-2148-7-190-S1.pdf]

Probabilistic model

The following derivation is based on a series of N independent tests. In our experiment N = 22 (for the 22 different CR1 subtypes). Each test can have N results. In general, the quantity of results can be from 1 to N. However, if any of the results at special time points are impossible, we shall consider their probabilities equal to 0. That is because the probabilities of these results depend on the time point of the test, as some retroposons (CR1 subtypes) evolved later than others and the timeframes of activity differ from subtype to subtype. This simply means that the oldest subtypes, after their inactivation, could not integrate into the youngest, because these did not yet exist at the point of their activity.

We designate $p_{i,j}(t)$ as the probability of the result j for experiment of the i-th series at time point t (i , $j \in \{1,2...N\}$, $t \in T_i = \{\tau_1^i, \tau_2^i, ..., \tau_{n_j}^i\}$). T_i is the set of time points in which the tests are performed. In other words we define for a time point t the probability of the result of a CR1 element i (one of the 22 subtypes) having inserted into the host CR1 element j (one of the remaining 21 subtypes).

Given that for any $t \in T_i$ $\sum_{j=1}^{N} p_{i,j}(t) = 1$

we assume, that if $i \neq j$, then $p_{i,j}(t)N << 1$ (accordingly, $p_{i,i}(t)$ is close to 1).

In the experiment we observe the values $m_{i,j}$ of the random variables $\mu_{i,j}$ - the quantities of approaches of a result j in the i-th series.

We consider $n_i = \sum_{j=1}^{N} m_{i,j}$ as the quantity of tests in the i-th series.

We define $\eta^j(t)$ as the quantity of elements of the subtype j (potential hosts) that are present at the time point t. By including the biological fact that elements of a subtype j «appear» (first evolve and than distribute) at time points $\tau_1^j, \tau_2^j, ..., \tau_{n_j}^j$, we obtain:

$$\eta^j(t) = \begin{vmatrix} 0, \ t \le \tau_1^j \\ \dots \\ k, \ \tau_k^j < t \le \tau_{k+1}^j \\ \dots \\ n_j \ \ \tau_{n_j}^j < t \end{vmatrix} \tag{1}$$

Thus the ratio

$$F^j(t) = \frac{\eta^j(t)}{n_j} \tag{2}$$

can be considered as an empirical distribution function of a random variable τ^j. In other words this function gives the ratio of CR1 elements of a subtype *j* that, at time point *t*, already exist and the number of CR1 elements of a subtype *j* that exist over the observation time.

The probability $p_{i,j}(t)$ (at $i \ne j$) is considered to be proportional to $\eta^j(t)$:

$$p_{i,j}(t) = \alpha \cdot \eta^j(t) \text{ under the condition } t \in T_i. \tag{3}$$

Further we assume that $\alpha \cdot n_j << 1$. Under this assumption it is possible to prove that for the *i*-th series at $n_i \to \infty$ the Poisson distribution can be applied:

$$\mathrm{P}(\bigcap_{j \ne i} \{\mu_{i,j} = x_j\}) = \prod_{j \ne i} \frac{a_{i,j}^{x_j}}{x_j!} \cdot e^{-a_{i,j}}, \tag{4}$$

where

$$a_{i,j} = \sum_{t \in T_i} p_{i,j}(t) = \sum_{k=1}^{n_i} p_{i,j}(\tau_k^i). \tag{5}$$

Accordingly, for all *N* series

$$\mathrm{P}(\bigcap_{i=1}^{N} \bigcap_{j \ne i} \{\mu_{i,j} = x_{i,j}\}) = \prod_{i=1}^{N} \prod_{j \ne i} \frac{a_{i,j}^{x_{i,j}}}{x_{i,j}!} \cdot e^{-a_{i,j}}. \tag{6}$$

According to (5) and (3) it follows that

$$a_{i,j} = \alpha \cdot \sum_{k=1}^{n_i} \eta^j(\tau_k^i). \tag{7}$$

The sum can be written as Stieltjes integral:

$$a_{i,j} = \alpha \cdot \int_{-\infty}^{\infty} \eta^j(t) \cdot d\eta^i(t) \ , \tag{8}$$

Inserting equation (2), we get the following:

$$a_{i,j} = \alpha \cdot n_i \cdot n_j \cdot \int_{-\infty}^{\infty} F^j(t) \cdot dF^i(t) \qquad . \tag{9}$$

As an approximation for $F^j(t)$ we chose the normal distribution with the parameters t_j, σ_j

$$F^j(t) = F\left(\frac{t - t_j}{\sigma_j}\right) , \tag{10}$$

where $F(x) = \frac{1}{\sqrt{2\pi}} \int_{-\infty}^{x} e^{-\frac{y^2}{2}} dy$ is the standard function of the normal distribution.

Then, with (9), we receive

$$a_{i,j} = \alpha \cdot n_i \cdot n_j \cdot F_{i,j} \ , \tag{11}$$

where

$$F_{i,j} = F\left(\frac{t_i - t_j}{\sqrt{\sigma_i^2 + \sigma_j^2}}\right) . \tag{12}$$

Thus the problem is reduced to the search for estimations of the unknown parameters $\alpha, t_1 ... t_N, \sigma_1 ... \sigma_{N.}$.

To estimate these parameters, we use a maximal likelihood method. Replacing, in (6), $x_{i,j}$ to $m_{i,j}$ and lowering multipliers, not dependent on estimated parameters, in view of (11), we receive the function of likelihood

$$l = l(\alpha, t_1 ... t_N, \sigma_1 ... \sigma_N) = \prod_{i=1}^{N} \prod_{j \neq i} \left(\alpha \cdot F_{i,j}\right)^{m_{i,j}} \cdot e^{-\alpha \cdot n_i \cdot n_j \cdot F_{i,j}} \ . \tag{13}$$

Accordingly, the logarithm of the function of likelihood is equal to

$$\ln(l) = \sum_{i=1}^{N} \sum_{j \neq i} \left(m_{i,j} \cdot \ln(\alpha \cdot F_{i,j}) - \alpha \cdot n_i \cdot n_j \cdot F_{i,j} \right). \tag{14}$$

Combining $F(-x) = 1 - F(x)$ and (12) we get $F_{i,j} + F_{j,i} = 1$.

So the sum $\sum_{i=1}^{N} \sum_{j \neq i} n_i \cdot n_j \cdot F_{i,j} = \sum_{i=1}^{N-1} \sum_{j=i+1}^{N} n_i \cdot n_j$ does not depend on any parameters.

This sum we designate as n_0:

$$n_0 = \sum_{i=1}^{N-1} \sum_{j=i+1}^{N} n_i \cdot n_j \ . \tag{15}$$

We also suppose

$$m_0 = \sum_{i=1}^{N} \sum_{j \neq i} m_{i,j} \ . \tag{16}$$

Thus it is possible to describe (14) in the form of

$$\ln(l) = \sum_{i=1}^{N} \sum_{j \neq i} m_{i,j} \cdot \ln(F_{i,j}) + m_0 \cdot \ln(\alpha) - n_0 \cdot \alpha \ . \tag{17}$$

Equating a partial derivative to zero

$$\frac{\partial \ln(l)}{\partial \alpha} = \frac{m_0}{\alpha} - n_0 \ ,$$

we receive an estimation for α :

$$\tilde{\alpha} = \frac{m_0}{n_0} \tag{18}$$

(for our experimental data we have received $\tilde{\alpha} = 1.003 \cdot 10^{-7}$).

$F_{i,j}$ does not change under the replacement:

$$\begin{array}{l} \sigma_i \to h \cdot \sigma_i \\ t_i \to h \cdot t_i + t_0 \end{array} .$$

Therefore it is possible to limit to a condition

$$\sum_{i=1}^{N} t_i = 0 .$$

Concerning parameters σ_i we have accepted that these are proportional to *n* (in particular $\sigma_i = n_i$).

The estimations of the other parameters we derive by maximizing ln(l), using the program *MathCad (Mathsoft Engineering & Education)*.

Application in this study:

We wrote a computer script to obtain the data of the TinT matrix (Table S1). The probabilistic model was then used to calculate the peaks of the activity distribution for each element on a relative timescale.

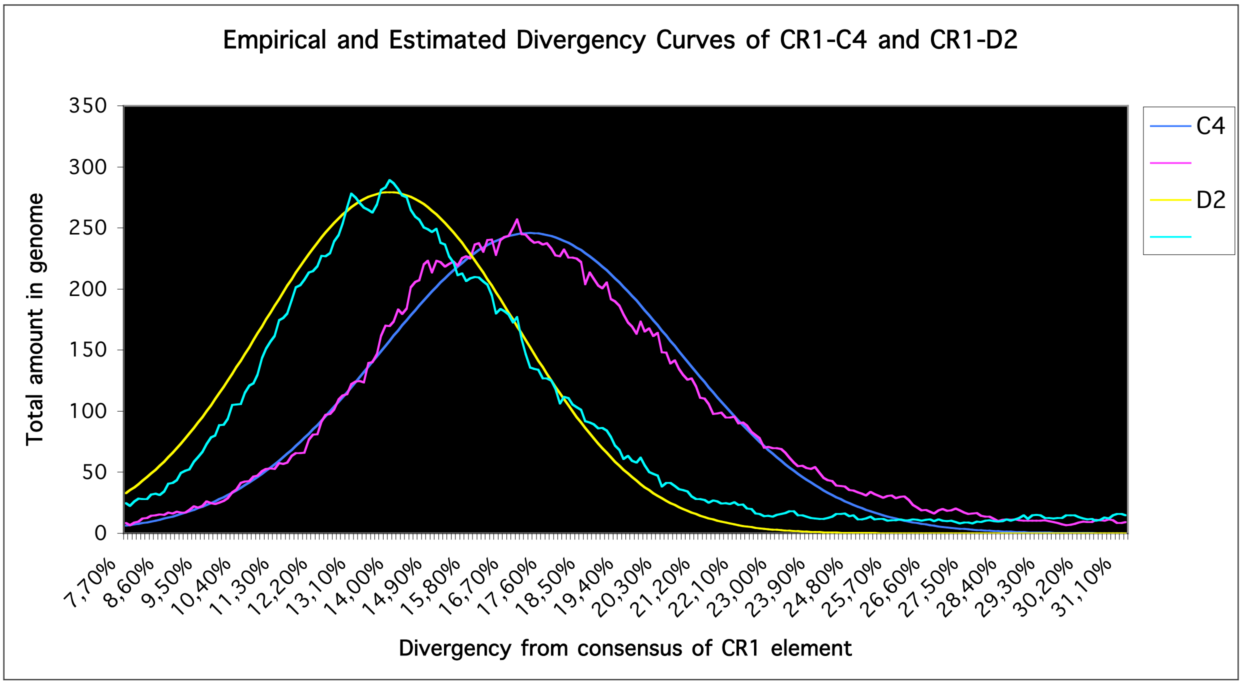

Figure S1. Examples of divergency distributions in two CR1 subtypes. The level of divergency from the consensus of a CR1 subtype is plotted against the total amount of copies in the chicken genome. The empirical divergency distribution of each CR1 subtype is approximated by the normal distribution (as it was also shown for *Alu* elements by R Mills, E Bennett, S Devine: Poster 47: A Positional Approach to Classifying Transposons. In: *FASEB Meeting on Mobile Elements in Mammalian Genomes*; Tucson, 2007)

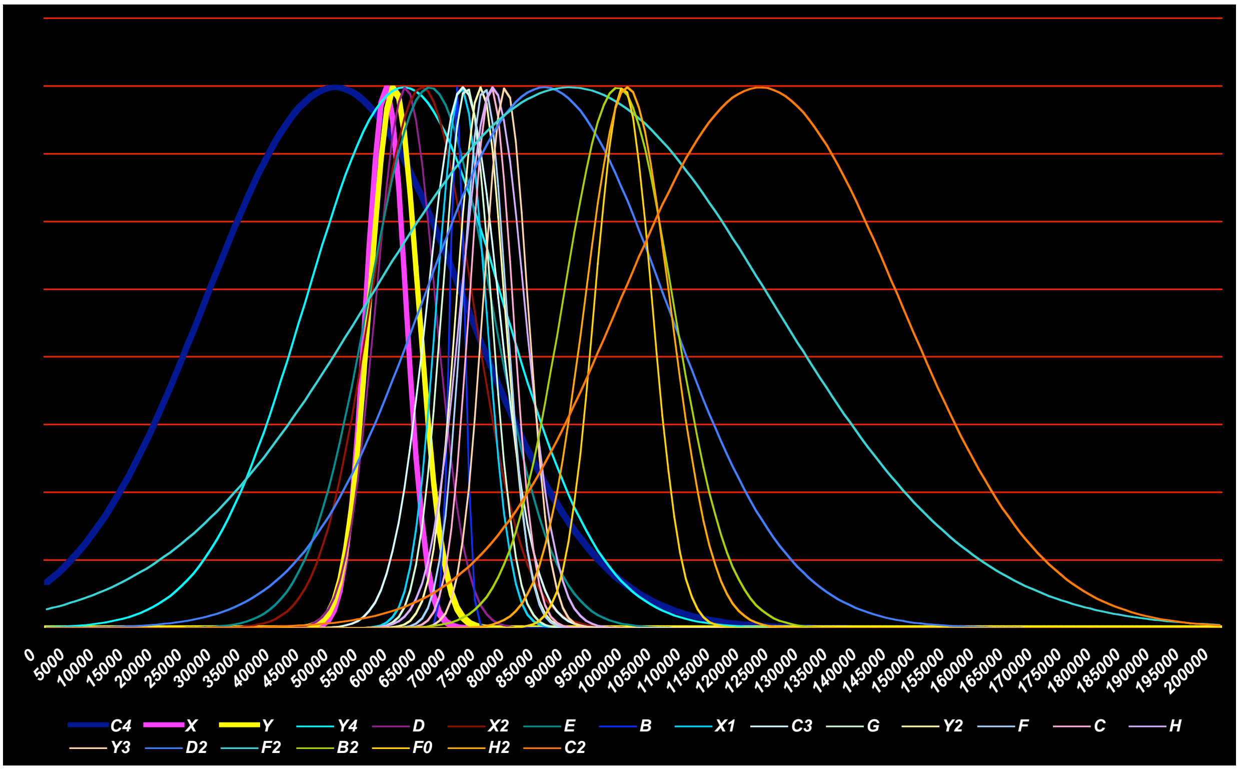

Figure S2. Activity distribution for each element on a relative timescale.

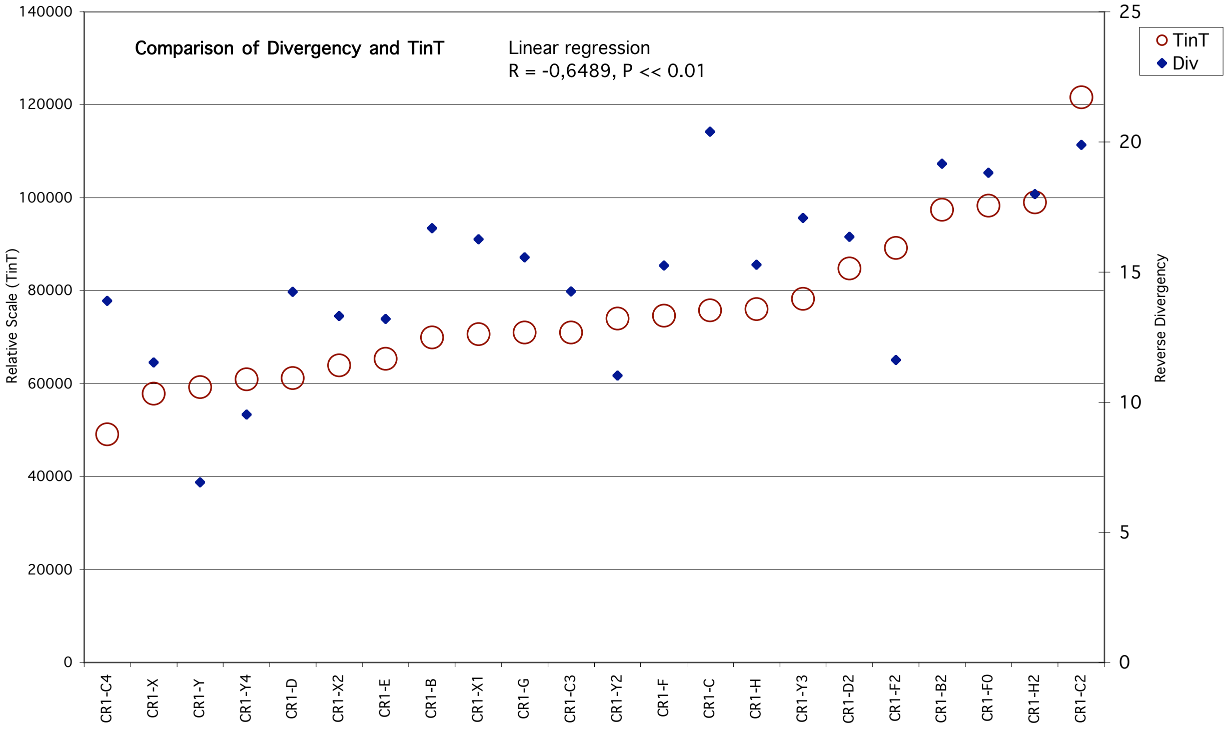

Figure S3. Comparison of Divergency level and relative TinT timescale.

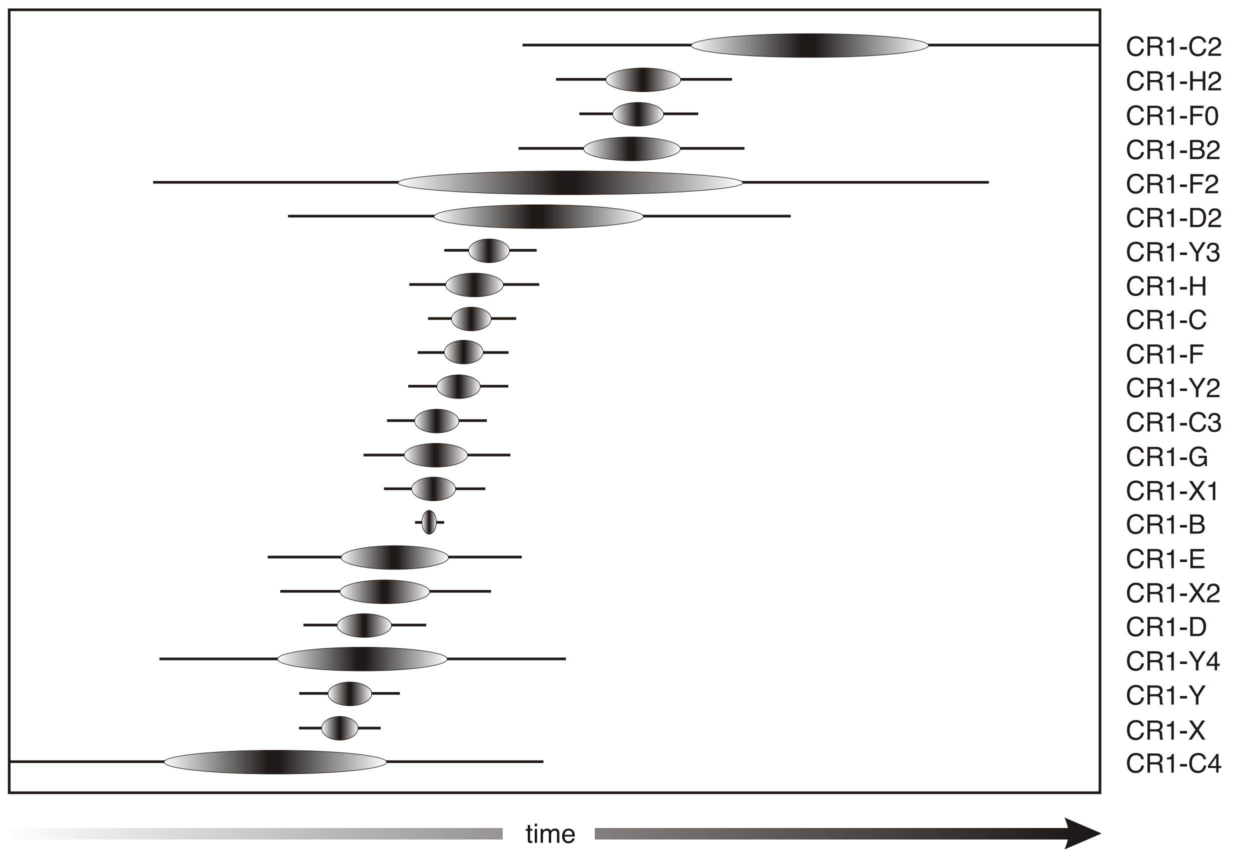

Figure S4. Normalized relative activity periods for the 22 Cr1 subtypes of the chicken genome. Ovals represent the 50% activity distribution with the median position in black. Horizontal lines indicate the 90% activity distribution of each element. The relative time axis is given at the bottom.
