# Supplementary material for: Waves of genomic hitchhikers shed light on the evolution of gamebirds (Aves: Galliformes)
Source: BMC Evol Biol. 2007 Oct 9;7:190. doi: 10.1186/1471-2148-7-190 (PMC2169234; doi:10.1186/1471-2148-7-190)
Supplement: Additional file 2 — contains a full presence/absence table of the phylogenetic analyses and a table of the PCR primers used to amplify informative loci. [file 1471-2148-7-190-S2.pdf]

Marker	1a	2a	2b	3a	3b	3c	3d	3e	3f	3g	3h	4a	5a	5b	5c	5d	5e	6a	6b	7a	8a	8b	9a	9b	10a
CR1-	E	Y2	X2	F2	X2	F2	Y4	X2	D2	D2	D2	H2	C2	B2	B2	C	(GGLTR4a)	B2	C	C	B2	B2	(GGERV-18LTR)	(GGERV-18LTR)	D2
Taeniopygia guttata	-																								
Anas crecca	+	-		-	-		-	-	-	-	-	-		-	-	-	-	-	-	-			-	-	
Cairinia moschata		-		-	-			-						-	-		-	-	-	-			-	-	
Alectura lathami	+	-	-	-	-	-	-	-	-		-	-	-	-	-	-	-	-	-	-	-		-	-	-
Crax fasciolata	+	+	+	-	-	-	-	-	-	-	-	-	-	-		-	-	-	-	-	-		-	-	+
Crax alecotor		+	+		-	-	-	-				-	-	-	-	-	-	-	-		-		-	-	+
Numida meleagris	+	+	+	+	+	+	+	+	+	+	+	-	-	-	-	-	-	-	-	-	-	-	-	-	-
Callipepla squamata		+	+	+	+	+	+	+		+	+	+	-	-	-	-	-	-	-	-	-	-	-	-	-
Colinus virginianus		+	+	+	+		+	+				+	-	-	-	-	-	-	-	-	-	-		-	-
Rollulus rouloul		+	+	+	+	+	+					+	+	+	+	+	+	-	-	-	-	-	-	-	-
Gallus gallus	+	+	+	+	+	+	+	+	+	+	+	+	+	+	+	+	+	+	+	-	-	-	+	+	-
Gallus lafayettii		+	+	+	+		+	+				+		+	+	+	+	+	+	-	-	-	+	+	-
Coturnix japonica		+	+	+	+	+		+				+	+	+	+	+	+		+		-	-	-	-	-
Afropavo congensis		+	+	+	+	+	+					+		+	+	+	+		+	+	-	-	-	-	
Pavo cristatus		+	+	+	+	+		+				+		+	+	+	+		+	+	-	-	-	-	
Pavo muticus		+	+	+	+	+	+	+				+	+	+	+	+	+		+	+	-	-	-	-	-
Tragopan caboti		+	+	+	+	+		+				+		+	+	+	+		+	+	+	+	-		
Perdix perdix		+	+	+	+	+	+	+				+	+	+	+	+	+	+	+	+	+	+	-		-
Chrysolophus amherstiae		+	+	+	+	+	+	+						+	+		+	+	+	+	+		-	-	
Chrysolophus pictus		+	+	+	+	+	+	+				+	+	+	+	+	+			+	+	+	-	-	-
Tetrao tetrix		+	+	+	+	+	+	+				+	+	+	+	+	+	+	+	+	+	+	-	-	-
Tympanuchus cupido		+	+	+	+	+	+	+				+	+	+	+	+	+		+	+	+	+	-	-	-
Meleagris gallopavo		+	+	+	+	+	+	+				+	+	+	+	+	+	+	+	+	+	+	-	-	-

Table S2. Presence (+) and absence (-) patterns. 1a-10a correspond to the analyzed markers. Row 2 denotes the CR1 subtypes or LTR elements (in brackets) present in the (+) state. For empty cells no data was obtained.

Table S3. Oligonucleotides used for PCR amplifications

primer	(5'-3')
1a-F	TGCCCATCTTGATGATAATGAGAG
1a-R	TCTCGCCTGCAATGTAAGTGAC
2a-F (5b)	CATTCCAGTGTCTGATGAAGCAG
2a-R (5b)	GTCAGAAGTAGCCACCATAGACAC
2b-F (8a)	GGAGGGACCTGGCAGCAC
2b-R (8a)	CAACACCGTGGACAGCATTG
3a-F	AAAGTTCGCTACGTGAAGCTG
3a-R	CTTCCTTGGTTTTCTCATCCAC
3b-F	AAGGTGACCCAGGTATTGAAG
3b-R	TTCTCACCTTTTCTCCTTTCAG
3c-F	CCACCACAGGGAGCTTCAG
3c-R	GGTTTCAACAAATCTCCTGGAC
3d-F	CCTCACAGCGAGTAACCATGAC
3d-R	GAACCAAGAAAATACATTGCTGC
3e-F	GGCTTAATGATTTCATTCAGAAG
3e-R	ATTTCCAAGATAGATTGAACTTCAG
3f-F	CTCTAACAATGAGCGAGAAGCG
3f-R	AGGTGATCCAGTTCCAGCATATC
3g-F	GTGACAGGAGTCCAGGTGATCTG
3g-R	GGAGCCACCACTGTGATGCAG
3h-F	CACGTGGAGATGAAGCAGAAC
3h-R	CTCCAGGTAGTTGTAGCCTTCATAG
4a-F	CATCAGACACCTCCTGGGTCAT
4a-R	GGCTGCTCAGCTTGCTGAC
5a-F (10a)	ATACCGCCTGTTCACACTGCT
5a-R	CGTACATTGCAAAGGATATCACAG

(10a)	
5a-Fa (10a)	TCATAGATGAGCTGAAACAG
5a-Ra (10a)	GAAATCATCGATTTGTGG
5c-F	GAGTTGGAGTGGTGATCGAAGC
5c-R	TTTTCTGTACCCCACGCATTAC
5c-Fa	GTGATCGAAGCTACGTAAG
5c-Ra	TTACCATACACATATGCCTAG
5d-F	TGACACTGTTTTATGTACACATCAAC
5d-R	TCCAGAATGAGAGAAGTAGCAGC
5e-F	CCAGTAATCCAGGATCTCTGTCAC
5e-R	GGACTATCCGTGACAAGTTTGC
6a-F	CGTGTATTTCTGGTGCACAGC
6a-R	ATGGTTTGCTGACAGCTATGC
6a-Fa	GAGTAAAATGACATGATTGC
6a-Ra	AGAAGCCATACGGATCTC
6b-F	CTACAGTAACACTCAGCATGTCTGC
6b-R	GTCCCATATCAGCAATCTTGTACTC
7a-F	GCAGGCACAGTTGGTAAAG
7a-R	TGATTCATTCGTCTCAGCTC
7a-Fa	CTGACTCTGAATTGGTCTCAG
7a-Ra	TATCCAAAGACCATGACCG
7a-Fb	TCCAGAATTGCAGTAGCTG
7a-Rb	AGCCTTCTGAGCCAATTG
8b-F	CCGTGAGCGCAACAAGACG
8b-R	CGGTGATGCCAGAGAACTTCTC
9a-F	TGCATAATAAGTCATCTGAGCTTC
9a-R	CGTCAAGCAGAGATTACCAGAC
9b-F	TCTGGCTTCTTTTCTAATAGCAATG
9b-R	TGCTGATTTTTGAAAACTTGATAGA

Primer names correspond to markers in Table S2. (F) denotes forward primers, (R) denotes reverse primers.
